# Incidence, accuracy, and barriers of diagnosing healthcare-associated infections: a case study in southeast Iran

**DOI:** 10.1186/s12879-023-08122-1

**Published:** 2023-03-21

**Authors:** Naser Nasiri, Ali Sharifi, Iman Ghasemzadeh, Malahat Khalili, Ali Karamoozian, Ali Khalooei, Amin Beigzadeh, AliAkbar Haghdoost, Hamid Sharifi

**Affiliations:** 1 Department of Biostatistics and Epidemiology, School of Public Health, Kerman University of Medical Sciences, Kerman, Iran; 2 HIV/STI Surveillance Research Center, and WHO Collaborating Center for HIV Surveillance, Institute for Futures Studies in Health, Kerman University of Medical Sciences, Kerman, Iran; 3 Department of Ophthalmology, Shafa Hospital, Afzalipour School of Medicine, Kerman University of Medical Sciences, Kerman, Iran; 4 Modeling in Health Research Center, Institute for Futures Studies in Health, Kerman University of Medical Sciences, Kerman, Islamic Republic of Iran; 5 Social Determinants of Health Research Center, Institute for Futures Studies in Health, Kerman University of Medical Sciences, Kerman, Iran; 6 Sirjan School of Medical Sciences, Sirjan, Iran

**Keywords:** Incidence rate, Healthcare-associated infections, Surveillance system, Accuracy

## Abstract

**Background:**

Healthcare-associated infections (HAIs) are a threat to patients. Accurate surveillance is required to identify and prevent HAIs. To estimate the incidence rate, report the accuracy and identify the barriers of reporting HAIs using a mixed-method study.

**Methods:**

In this quantitative study, we externally evaluated the incidence rate and accuracy of the routine surveillance system in one of the main hospitals by an active follow-up of patients from September to December 2021. We used in-depth interviews with 18 experts to identify the barriers of the routine surveillance system.

**Results:**

Among 404 hospitalized patients, 88 HAIs were detected. The estimated rate of HAIs was 17.1 (95% Confidence Intervals 95: 14.1, 21.1) per 1000 patient-days follow-up. However, in the same period, 116 HAIs were reported by the routine surveillance system, but the agreement between the two approaches was low (sensitivity = 61.4%, specificity = 82.6%, negative predictive value = 89.7%, and positive predictive validity = 46.5%). The minimum and maximum positive predictive values were observed in urinary tract infection (32.3%) and surgical site infection (60.9%). The main barrier of reporting HAIs was lack of cooperation in reporting HAIs by infection control link nurses and laboratory supervisors.

**Conclusions:**

The discrepancy between the longitudinal study findings and the routine surveillance might be related to the inaccessibility of the surveillance system to clinical information of patients. In this regard, decreasing the barriers, increasing the knowledge of infection control nurses and other nurses, as well as the development of hospital information systems are necessary.

## Introduction

“Healthcare-associated infections (HAIs) can happen in any healthcare facility, including hospitals, ambulatory surgical centers, end-stage renal disease facilities, and long-term care facilities [1].” HAIs are a big concern in treating some diseases [2], and pose a threat to patients' health and worldwide safety [3]. HAIs are a major cause of mortality [4], hospitalization [5], excess length of hospital stay [4], long-term disability, increased microorganism resistance to antimicrobial agents, increased costs for patients and family members [6], and health-related costs [7]. However, hospitalized patients are at a higher risk for HAIs, but the burden of HAIs is higher than other communicable diseases. In a study in 2016, the burden of HAIs was calculated to be 501 Disability Adjusted Life Years (DALYs) per 100,000 general populations in European Union and European Economic Area [8].

The incidence rate of HAIs varies from 2.5 per 1000 patient-days [9] to 28.15 per 1000 patient-days, worldwide [10]. In Iran, the incidence rate of HAIs was reported as 7.41 per 1000 patient-days [11]. HAIs can cause various problems in patients, including lower respiratory tract infection that raises mortality, and surgical site infection leads to more extended hospitalization and increased therapy costs [12]. It is estimated that 90% of the burden of the HAIs is related to ventilator-associated pneumonia (VAP), bloodstream infection (BSI), urinary tract infection (UTI), and surgical site infection (SSI) [8].

A standardized surveillance system is an essential strategy for controlling and reducing HAIs [12]. Iranian nosocomial infection surveillance (INIS) system was established in 2006 and expanded in 2010 [11, 13]. This system collects the required data passively using an online approach from all hospitals in Iran. The main approach for detecting HAIs in routine surveillance is the clinical manifestations. Collaboration between a nurse, a microbiologist, and an infection control team is essential in the Iranian surveillance system to diagnose HAIs [14]. According to the Ministry of Health and Medical Education of Iran, HAIs were detected twice as much in 2016 (1.32%) than in 2006 (0.6%) [15], and this could be related to the establishment of the surveillance system. Although the detection of HAIs has increased in recent years, the validity of the surveillance system has not been well studied [13]. Evidence for HAIs diagnosis may be challenging to obtain in passive surveillance, resulting in misclassification and underreporting [6] because clinical signs and symptoms are typically unavailable from medical records [16]. Regarding the importance of evaluating the available surveillance system, this study aimed to estimate the incidence rate of HAIs in general and in different types of HAIs and compare the findings with the reported incidence rate by the surveillance system. Moreover, the accuracy of reported HAIs was estimated, and the barriers of detecting HAIs were studied.

## Methods

### Study setting and design

To assess the incidence rate and the accuracy of HAIs diagnosis, we designed a 3-month longitudinal study from 23th September to 21th December 2021, with daily follow-ups. In parallel, we compared the incidence of HAIs using data extracted from the routine surveillance during the same period in a tertiary-care teaching hospital, Afzalipour hospital, in Kerman city, southeastern Iran. Furthermore, we conducted a qualitative study in 18 hospitals in different cities to identify the barriers of HAIs diagnosis.

### Part 1: quantitative study

In the longitudinal study, we included patients from six wards among 24 active wards in the hospital, based on disease variety, ward turnover, and the variety of HAI reports, including internal medicine, surgery intensive care unit (ICU), gastrointestinal diseases, pulmonary diseases, general surgery, and women^’^s surgery. We included all the admitted patients with a central venous catheter (CVC), mechanical ventilation, or Foley catheter from four wards (internal medicine, surgery ICU, gastrointestinal diseases, and pulmonary diseases). Patients in two wards (general surgery and women’s surgery) were not followed up for device infection because Foley catheters were fixed in the patients at the time of operation. Severe patients in two wards were transformed into surgery ICUs. We also added two wards, general surgery, and women's surgery, to detect SSIs because these two wards were responsible for most of the SSIs reported in the hospital.

Follow-ups of patients were started after admission to the ward and ended if they were discharged or died. A trained and experienced nurse (NN) referred to six wards daily and asked the nurses who were in charge of infection control and other staff about HAIs. He followed up patients and asked them or their relatives and the responsible nurse to look for signs or symptoms of infection. He also recorded the mode of ventilators set according to the bedside chart and observed the mode on the ventilator screen. We excluded all infections acquired in the community before the admission and secondary infection events in BSI.

In order to reach the objectives of the study, these data were recorded during the follow-up period: the admission code, hospitalization history, cause of the hospitalization, signs of infection, urine catheter date, CVC date, endotracheal tube date, registered vital signs, clinical symptoms, laboratory findings, surgical intervention record, positive end-expiratory pressure** (**PEEP) of the ventilator, and FiO2 daily. Also, we registered demographic data (age, sex), comorbidities, disease history, date of hospitalization, and discharge or death date.

Definition of HAIs in the longitudinal study and routine surveillance was based on the standard definitions of INIS [15]. HAIs were diagnosed according to the case definition of INIS and consulted with an infectious diseases specialist (IG) or according to the therapeutic physician’s opinion. The definition of included HAIs is presented in Table 1. Central Line-associated Bloodstream Infection (CLABSI) is one of the four main causes of HAIs, but in our study, it is not investigated because, up to now, we do not have a detection method in use. In particular, we or some of our hospitals do not apply. The Infectious Diseases Society of America published recommendations for the diagnosis and treatment of CLABSI. They describe preferential culturing of catheter tips and avoidance of broth culturing techniques and define the interpretation of roll plate (> 15 CFU from a 5 cm segment) and sonication techniques (> 102 CFU) when assessing for colonization. CLABSI diagnosis can be made when culture results identify the same organism in at least the culture obtained as a peripheral stick and from a culture of the catheter tip. If the catheter is left in place, the diagnosis can be made if there are two blood samples being drawn (one from the catheter and one from a peripheral stick) that meet specific criteria for quantitative blood cultures or differential time to positivity [17, 18]. To assess the discrepancy between the report of routine surveillance and the result of the longitudinal study, we referred to the hospital archive and reviewed the patients’ records.

**Table 1:** Definitions of any types of healthcare-associated infections to measure the incidence rate and reporting accuracy

Category	Subcategory	Definition
SSI^ф^	Superficial Incisional	Infections 30 days after the operation and infection involves only skin and subcutaneous tissue, and at least one of the following criteria: **I.** Purulent drainage from the superficial incision. **II.** Culture-positive from the superficial incision. **III.** At least one of the following signs or symptoms of infection: pain or tenderness, localized swelling, redness, or fever, and superficial incision that is deliberately opened by the surgeon and not cultured. **IV.** Diagnosis of superficial incisional SSI by the surgeon or attending physician
	Deep Incisional	Deep SSI infection is identified by infections 30 or 90 days after the operation. It involves deep soft tissues (e.g., fascial and muscle layers), and the patient has at least one of the following: **I.** Purulent drainage from the deep incision. **II.** A deep incision spontaneously dehisces or is deliberately opened by a surgeon and is culture-positive or not cultured. The patient has at least one of the following signs or symptoms: a. Fever above 38 °C (100.4°F) or localized pain or tenderness. b. The evidence of abscess or infection in direct examination or by histopathologic or radiologic examination
SUTI^1^	CAUT^ɰ^	**I.** The patient was fitted with a Foley catheter for two days, **II.** And at least one of the following symptoms or signs involved: fever above 38 °C (100.4°F), suprapubic tenderness, costovertebral angle tenderness or pain, urinary incontinence, frequent urination, urine urgency, **III.** And positive culture
	non-CAUTI^ʤ^	**I.** Patients have no Foley catheter, **II.** And at least one symptom, and sign fever above 38 °C (100.4°F), suprapubic tenderness, costovertebral angle tenderness or pain, urinary incontinence, frequent urination, urine urgency **III.** And positive culture
BSI^2^		Definite: **I.** At least a positive culture with well-known pathogen BSI, **II.** And non-secondary BSI
VAP^ϯ^		VAP is a definite combination of laboratory and clinical findings as **I.** After at least 2 days stability of the patient’s condition with ventilation, at least raise 0.20 in min FiO2 for a least 2 days, or at least grow three cm H_2_O in PEEP for at least two days. **II.** Fever above 38 °C (100.4°F), leukopenia (WBC 4000/mm^3^ or less) or leukocytosis (WBC 12,000/mm^3^ or more), and began new antibiotic at least four days. **III.** Purulent respiratory secretion IIII, and positive culture

^ф^SSI: surgical site infection; ^1^SUTI: Symptomatic urinary tract Infection; ^ɰ^CAUTI: catheter-associated urinary tract infection; ^ʤ^non-CAUTI: non-catheter-associated urinary tract; ^2^BSI: bloodstream infection; ^ϯ^VAP: ventilator-associated pneumonia

### Data management and analysis

After completing the follow-up period, another author (AK) was referred to the hospital’s infection control center and received data on the routine surveillance systems for each HAI. The admission code was used to link data from the routine surveillance system and the longitudinal study. We used hospital information systems (HIS) data to reduce missing data and confirm data validity using laboratory and pharmacy information.

Data were analyzed using descriptive statistics (proportion and percentage for categorical data, mean and standard deviation for continuous data). Also, the incidence rate of HAIs in general and for each specific HAIs (per 1000 patient-days), and their 95% Confidence Intervals (CI) were calculated. The Chi-square test was used to compare proportions in two approaches (longitudinal study and routine surveillance). Kappa, sensitivity, specificity, positive predictive value (PPV), and negative predictive value (NPV) were also calculated. All analyses were calculated by Stata software version 14.2 (StataCorp, College Station, TX, USA).

### Part 2: qualitative study

To assess the barriers of the detection of HAIs, we carried out a qualitative study. In this study, using convenient sampling, 18 infection control experts in different hospitals around the country were recruited for interview. The interview was conducted in a private, public, or military hospital with more than 100 active beds. They were asked about their experiences in controlling infection and their opinion on barriers of detection and registration of HAIs. Data were obtained using semi-structured interviews in 20–40 min. We obtained explicit consent from participants to record their interviews with an audio recording device. Data saturation was reached after interview 14.

The interviews were analyzed using content analysis. The procedures applied were as follows: after implementing the interviews by the first author, they were transcribed verbatim by two separate authors. The credibility, transferability, dependability, and confirmability of the interviews were considered and confirmed [19, 20]. Each interview was read to get a general understanding and meaning units were identified and condensed to be labeled with codes. Then, codes were classified into subcategories and categories (the primary codes), and finally the underlying meaning was extracted. MAXQDA software version 10 (VERBI Software; Udo Kuckartz, Berlin, Germany) was used to analyze the data.

## Results

**Fig. 1:**
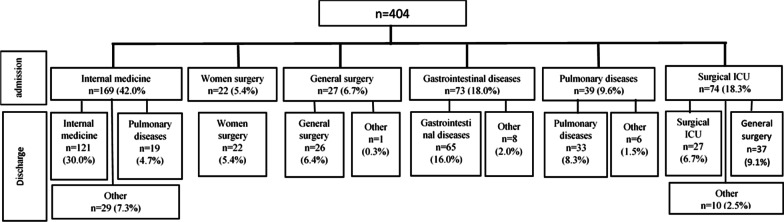
Flow chart of the included participants to assess the incidence of healthcare-associated infections (n = 404)

Among 404 enrolled patients, 42.0% (n = 169) were in internal medicine ward, 18.3% (n = 74) were in surgery ICU ward, 18.0% (n = 73) were in gastrointestinal diseases ward, 9.6% (n = 39) were in pulmonary ward, 6.7% (n = 27) were in general surgery ward, and 5.4% (n = 22) were in women^’^s surgery ward (Fig. 1).

The mean age of the patients was 58.5 ± 1.05 years, and 51.7% (n = 209) were male. The mean length of stay at the hospital was 11.1 ± 0.5 days. The prevalence of comorbidities was: hypertension (37.0%), diabetes (22.0%), gastrointestinal diseases (16.8%), heart disease (16.0%), and cancer (14.5%). Totally, 26.6% (n = 106) of the admitted patients died during the follow-up. Mortality proportion among the patients with HAIs was 40.9% (n = 36 out of 88; 95% CI: 30.5, 51.9), and it was significantly higher than those without HAIs 22.2% (n = 70 out of 316; 95% CI: 18.1, 27.6; P_value = 0.001) (Table 2).

**Table 2:** Demographic, clinical, and hospitalization characteristics from patients included in the longitudinal study

Variable	Total, n (%) n = 404	VAP^ϯ^ n (%) n = 17	SSI^ф^ n (%) n = 29	BSI^2^ n (%) n = 27	UTI^1^ n (%) n = 15
Sex					
Male	209 (51.7)	8 (47.0)	6 (20.7)	13 (48.2)	6 (40.0)
Female	195 (48.3)	9 (53.0)	23 (79.3)	14 (51.9)	9 (60.0)
Hospital discharge					
Recovery	233 (58.4)	1 (5.9)	23 (79.3)	12 (44.4)	6 (40.0)
Discharges against medical advice	40 (10.1)	0 (0.0)	2 (6.9)	1 (3.7)	0 (0.0)
Death	106 (26.6)	15 (88.2)	2 (6.9)	12 (44.4)	7 (46.7)
Transport to another hospital	20 (5.0)	1 (5.9)	2 (6.9)	2 (7.5)	2 (13.3)
Start day of antibiotic prescription					
First day	191 (59.5)	12 (70.6)	16 (55.2)	17 (63.0)	7 (46.6)
Second days and above	130 (40.5)	5 (29.4)	13 (44.8)	10 (37.0)	8 (53.4)
Days with Endotracheal ventilation					
< 8 days	28 (56.0)	2 (11.8)	4 (100.0)	7 (70.0)	2 (28.6)
≥ 8 days	22 (44.0)	15 (88.2)	0 (0.0)	3 (30.0)	5 (71.4)
Number of urine catheter					
1	289 (91.7)	15 (88.2)	7 (70.0)	19 (82.6)	15 (80.0)
≥ 2	28 (8.3)	2 (11.8)	3 (30.0)	4 (17.4)	3 (20.0)
Mean ± SD					
Age (years)	58.5 ± 1.05	57.06 ± 4.78	40.0 ± 3.68	57.02 ± 5.07	57.3 ± 4.60
Admission duration (days)	11.11 ± 0.5	22 ± 2.95	13.48 ± 2.32	14.62 ± 2.5	20.9 ± 4.03
Duration of antibiotic Use in HAI patients (years)	12.26 ± 0.93	20.35 ± 2.65	10.38 ± 1.62	10.7 ± 1.6	16.53 ± 3.86

^ϯ^VAP: ventilator-associated pneumonia; ^ф^SSI: surgical site infection; ^2^BSI: bloodstream infection; ^1^UTI: urinary tract infection

Among 404 recruited patients in the longitudinal study, 88 HAIs (21.8%; 95% CI: 17.9, 26.1) were detected. The incidence rate of HAIs in the longitudinal study was 17.1 (95% CI: 14.1, 21.1) per 1000 patient-days follow-up. The incidence rate of SSI was 6.5, BSI was 6.1, VAP was 3.8, and UTI was 3.4 per 1000 patient-days (Table 3). Among the 404 recruited samples, 116 HAIs (28.7%; 95% CI: 24.3, 33.4) were detected in the same period by the routine surveillance system (incidence rate: 22.5; 95% CI: 18.8, 27.0) per 1000 patient-days.

**Table 3:** Incidence rate (per 1000 patient-days) of healthcare-associated infections according to longitudinal study and surveillance data

Variable	Longitudinal study	Surveillance data
	Incidence rate (95% CI^*^)	Incidence rate (95% CI)
HAIs^Ϯ^	17.1 (14.1, 21.1)	22.5 (18.8, 27.0)
VAP^ϯ^	3.8 (2.4, 6.1)	9.1 (6.6, 12.2)
SSI^ф^	6.5 (4.5, 9.4)	5.2 (3.4, 7.8)
BSI^2^	6.1 (4.2, 8.8)	4.9 (3.3, 7.5)
UTI^1^	3.4 (2.1, 5.6)	7.1 (5.1, 9.9)

^Ϯ^HAIs: healthcare-associated infections; ^ϯ^VAP: ventilator-associated pneumonia; ^ф^SSI: surgical site infection; ^2^BSI: bloodstream infection; ^1^UTI: urinary tract infection; *CI: Confidence interval

The sensitivity of routine surveillance system for detecting HAIs was 61.4% (95% CI: 56.8, 65.9). Also, it was 82.6% (95% CI: 79.1, 86.1) for specificity, 46.5% (95% CI: 41.9, 51.2) for PPV and 89.7% (95% CI: 86.8, 92.5) for NPV. The highest sensitivity was 76.5% (95% CI: 72.3, 80.6) for the reporting of VAP, and UTI was 66.7% (95% CI: 62.1, 71.3); however, the lowest sensitivity was 44.4% (95% CI: 39.6, 49.3) for the reporting of BSI, and SSI was 48.3% (95% CI: 43.4, 53.1). The highest PPV related to reporting of SSI was 60.9% (95% CI: 56.1, 65.6), and BSI was 54.5% (95% CI: 49.7, 59.4); however, the lowest PPV related to reporting of UTI was 32.3% (95% CI: 27.7, 36.8), and VAP was 32.5% (95% CI: 27.9, 37.1) (Table 4).

**Table 4:** Accuracy of surveillance data and disagreement between longitudinal study and surveillance data

No. of cases according to surveillance system	No. of cases detected according to the longitudinal study									
	Non HAIs	HAIs^Ϯ^	Not VAP	VAP^ϯ^	Not SSI	SSI^ф^	Not BSI	BSI^2^	Not UTI	UTI^1^
No	295	34	360	4	366	15	367	15	368	5
Yes	62	54	27	13	9	14	10	12	21	10
Sensitivity % (95% CI^*^)	61.4 (56.8, 65.9)		76.5 (72.3, 80.6)		48.3 (43.4, 53.1)		44.4 (39.6, 49.3)		66.7 (62.1, 71.3)	
Specificity% (95% CI)	82.6 (79.1, 86.1)		93.1 (90.6, 95.5)		97.6 (96.1, 99.1)		97.4 (95.8, 98.9)		94.6 (92.4, 96.8)	
PPV^¶^% (95% CI)	46.5 (41.9, 51.2)		32.5 (27.9, 37.1)		60.9 (56.1, 65.6)		54.5 (49.7, 59.4)		32.3 (27.7, 36.8)	
NPV^ɠ^% (95% CI)	89.7 (86.8, 92.5)		98.9 (97.9, 99.9)		96.1 (94.2, 98.1)		96.1 (94.2, 98.1)		98.7 (97.5, 99.8)	
Kappa^ɰ^ (95% CI)	0.4 (0.30, 0.48)		0.42 (0.33, 0.51)		0.51 (0.41, 0.60)		0.46 (0.36, 0.55)		0.41 (0.31, 0.05)	

^¶^PPV: positive predictive value; ^ɠ^NPV: negative predictive value; ^ɰ^Kappa: Cohen’s kappa; ^Ϯ^HAIs: healthcare-associated infections; ^ϯ^VAP: possible ventilator-associated pneumonia; ^ф^SSI: surgical site infection; ^2^BSI: bloodstream infection; ^1^UTI: urinary tract infection; *CI: confidence intervals

All the interviewed ICNs were women; most of them were in the age range of 33–55 years and had 1–15 years of experience. The main barriers of identifying HAIs were: lack of collaboration from the infection control link nurses (ICLNs), lack of cooperation of laboratories, lack of motivation, prescription of antibiotics before culture, different responsibilities, the heavy workload of ICNs, and not having up-to-date knowledge (Table 5). Moreover, 11 out of 18 ICNs announced that the identification of HAIs was only based on the culture results registered in HIS.

**Table 5:** Barriers of diagnosing HAIs, according to an interview with 18 experts responsible for controlling infection

Theme (n)	Example
lack of collaboration of ICLNs*	“We must walk through the wards and ask one by one that the patients, for example, do not have fever today. Or, in the case of the patient who returned after surgery, why did he return? You did not report this culture, no, no, this one came from another hospital, this one came from another ward, staff did not report.”
Different responsibilities and heavy workload of ICNs**	“My infection control supervisor is not a full-time employee, I am a clinical supervisor, which means I have no free time as for infection control.”
lack of laboratory collaboration	“Lack of laboratory cooperation means, …. we monitor them every three months, it means that they really exist, but these are not reported.”
lack of motivation	We have been oppressed a lot … To be honest, we have little motivation, I was in the infection control group a lot, most of us have meetings … The least observed place is the infection control unit
Prescription of antibiotics before culture	“It is an overuse of antibiotics. The patient started antibiotics and then was sent for culture.”
lack of up-to-date knowledge	“Sometimes it means that the training was supposed to be a comprehensive training … which was a very incomplete training, that is, a book training… nosocomial infections are detectable after surgery. It is simpler …, but what about those three, respiratory infections and urinary tract infections? What do we intend to do? We do not have a set program.”

*ICLN: infection control link nurses; **ICN: infection control nurses

## Discussion

In this study, the incidence rate of HAIs was estimated at 17.1 per 1000 patient-days in the longitudinal study; however, this was 22.5 per 1000 patient-days for the routine surveillance system. However, the incidence rate of detection was higher in the routine surveillance, but the detection accuracy was low. In this regard, the sensitivity of the routine surveillance was 61.4%, the specificity was 82.6%, and the NPV was 89.7%. The main problem of routine surveillance was low PPV (46.5%). The lowest PPV was related to the detection of UTI (32.3%) and VAP** (**32.5%); however, the highest PPV was related to the detection of SSI (60.9%). The most significant challenges for detecting HAIs were lack of collaboration of ICLNs as well as lack of collaboration of laboratory supervisors.

In the longitudinal study, the incidence rate of HAIs was 17.1 per 1000 patient-days, and the most incident infection was related to SSI (6.5 per 1000 patient-days). The estimated incidence in this study was higher than estimates in Scotland (3.3 per 1000 patient-days) [9], Turkey (3.6 per 1000 patient-days) [21], China (3.6 per 1000 patient-days) [22]; however, it was lower than in Ethiopia (28.2 per 1000 patient-days) [10]. The incidence of HAIs in a study conducted by Iranian nosocomial infection surveillance in 2020 (incidence rate 7.41 per 1000 patient-days) was lower than the estimated incidence in this study [11]. The higher incidence rate of HAIs in this study compared to the estimated HAIs in the developed countries could be due to different reasons such as long length of hospital stay [22], various definitions, a low record of healthcare-associated infection in some regions, and a higher record of healthcare-associated infection in teaching hospitals [23]. As healthcare-associated infections impose a significant burden on the system and patients [23], valid data is necessary to track, prevent, and control HAIs [23, 24]. Increasing the accuracy of surveillance systems and conducting multicenter longitudinal studies are necessary to estimate the incidence of HAIs better.

However, the routine surveillance in the hospital detected more cases than the longitudinal study, but the accuracy of reporting HAIs was low. The significant problem in the accuracy of HAIs detection was related to the low PPV. In this regard, the longitudinal study confirmed less than half of the detected cases as HAIs. PPV in all kinds of HAIs was low in the routine surveillance; however, the lowest PPV was reported on the diagnosis of UTI and VAP. Low PPV could be related to some items, including detection of HAIs based on the culture and the lack of access to clinical signs and symptoms [16], and the precision of some infection indices (e.g., the presence of fever in patients) was low [25]. Empowering the routine surveillance system to diagnose and control HAIs at the national and hospital levels is necessary. The capacity of HAIs surveillance in diagnosis and control of HAIs is related to various factors, including hospital microbiology capacity, susceptibility testing, high staff turnover, the quality of patient medical records, and collaboration of ICLNs [16]. In Iran, laboratory culture tests are an important part of detecting and controlling infections; however, intersectoral collaboration, standard laboratories, and supplies are limited [26]. Surveillance requires information from emergency department reports, admission history, and physical reports, including signs, symptoms, bedside interventions, diagnostic imaging, physician impressions, general consulting reports, antimicrobial treatment, and physician impressions [27]. The surveillance system may have missed this information, resulting in an error in detecting HAIs [28]. A computerized surveillance system to detect respiratory infections and SSIs, as well as the use of technologies to gain access to patient signs, symptoms, interventions, and physician assessments, are required [27]. As a result, we recommend improving intersectoral collaboration and laboratory capacity and developing the HIS.

Lack of collaboration from ICLNs, and laboratory supervisors were the main barriers of the detection and control of HAIs in Iran. A study in Iran reported that poor intersectoral partnership was one of the barriers of controlling HAIs [26]. Microbiology reports and patient medical charts are required for case detection in HAIs surveillance [29]. For instance, patient medical records and microbiologic evidence of pulmonary parenchymal infection are critical for VAP diagnosis, and impact VAP incidence and outcome reports [30]. Moreover, if the primary case-finding method in SSI is only based on microbiology reports, the ICN may miss SSI or some cases detected as SSI, incorrectly [31]. Although the laboratory surveillance method has a high sensitivity, approximately one out of every four cases of HAIs classified using the laboratory method are not true HAIs [32]. Weaknesses in case finding [29], and the use of laboratory results alone [32], may cause misdiagnosis of HAIs [29]. So, some multidisciplinary interventions and a decrease of barriers are necessary to report and control HAIs [26]. Therefore, it is advisable that policymakers focus on detecting and removing the barriers of the surveillance system’s accuracy. This could improve the surveillance system’s ability to detect HAIs.

This study had three limitations. First, we collected data from a tertiary-care teaching hospital in southeast Iran, thus, generalizability of the findings to other hospitals in the country may be difficult. Second, patients were not followed up after discharge, so we might have missed some cases after discharge which may underestimate the reported incidence rate. Third, although antibiotic use is prevalent in hospitalized patients, some patients may not show the signs of infection, which could underestimate the HAIs.

## Conclusions

We found that the incidence rate of HAIs was 17.1 per 1000 patient-days. This indicates that 17.1 out of 1000 hospitalized patients daily showed a sign of HAIs. However, the estimated incidence rate based on the hospital’s routine surveillance system was higher (21.8 out of 1000 hospitalized patients daily) than active follow-up of patients in the longitudinal study. Still, the accuracy of detection HAIs was low in the routine surveillance system. The low PPV in the detection of HAIS was the main problem of routine surveillance in detecting HAIs in general and for each specific HAI. This discrepancy could be related to the differences in data collection methods, so routine surveillance in Iran depends on the collaboration from ICLNs and laboratory supervisors. We also found some barriers of misdiagnosis of the surveillance system, including, lack of collaboration from ICLNs, the high workload of ICNs, lack of laboratory cooperation, and lack of sufficient knowledge. To better control HAIs, empowering the current surveillance, focusing on local and global data, and minimizing barriers of recognizing and reporting HAIs is necessary.

## Data Availability

The datasets used and/or analysed during the current study are available from the corresponding author on reasonable request.
